# Importance of lactic acid bacteria in Asian fermented foods

**DOI:** 10.1186/1475-2859-10-S1-S5

**Published:** 2011-08-30

**Authors:** Sook Jong Rhee, Jang-Eun Lee, Cherl-Ho Lee

**Affiliations:** 1 Department of Food Technology, Korea University, Seoul, 136-701, Korea

## Abstract

Lactic acid bacteria play important roles in various fermented foods in Asia. Besides being the main component in *kimchi* and other fermented foods, they are used to preserve edible food materials through fermentation of other raw-materials such as rice wine/beer, rice cakes, and fish by producing organic acids to control putrefactive microorganisms and pathogens. These bacteria also provide a selective environment favoring fermentative microorganisms and produce desirable flavors in various fermented foods. This paper discusses the role of lactic acid bacteria in various non-dairy fermented food products in Asia and their nutritional and physiological functions in the Asian diet.

## Introduction

Fermentation is one of the oldest forms of food preservation in the world. Fermented dairy products and their microbial and functional characteristics have been widely studied. Most East-Asian fermented foods are non-dairy products featuring various other food raw-materials such as cereals, soybeans, fruits, and vegetables, as well as fish and other marine products. The importance of lactic acid bacteria in fermented non-dairy foods and beverages was reviewed previously in the early 1990s in an overview of the role of lactic acid bacteria in *kimchi*, fermented fish products and vegetable yogurts [1]. The role of lactic acid bacteria in rice wine/beer fermentation has also been reported [2]. Steamed bread such as *idli* from India and *puto* from the Philippines, which are made with rice, are fermented by *Leuconostoc*. Korean *sikhae* and Philippine *burong isda/dalag*, which are made by mixing salted fish and cereals, are also fermented in early stages by *Leuconostoc mesenteroides*[3,4]. *L. mesenteroides* initiates relatively rapid growth in various plant materials (vegetables and cereals) over a wide range of temperatures and salt concentrations in comparison with other lactic acid bacteria. During growth, *L. mesenteroides* produces carbon dioxide and acids, leading to modification of the environment as well as conditions that favor the growth of other lactic acid bacteria. Analogously, the various fermentation pathways initiated by *L. mesenteroides* include prominent roles for other lactic acid bacteria belonging to the genera *Lactobacillus* and *Pediococcus* at later stages.

The numerous fermented food products in Asia can be categorized into five groups: (1) fermented soybean products, (2) fermented fish products, (3) fermented vegetable products, (4) fermented bread and porridges, and (5) alcoholic beverages. Lactic acid bacteria are involved in all of these fermentations to a varying extent, having either positive or negative effects on the eventual product. In making soybean sauce and paste, souring is indicative of poor fermentation and should be avoided. It is caused by undesirable yeast contamination, which is related to the fact that soybeans are not favorable substrates for the growth of lactic acid bacteria. In the case of alcoholic fermentation, lactic acid bacteria generally deteriorate the quality of the products. However, in traditional cereal alcoholic fermentation, lactic acid bacteria during the initial stage of fermentation provide a favorable environment for later stage fermentations including alcohol production, thereby contributing to the characteristic taste and aroma of the beverage. In fermentation of vegetable-derived raw materials, lactic acid bacteria play a major role, and the optimum amount of acid production varies with the product type [5].

The advantages of acidic food fermentation are: (1) renders foods resistant to microbial spoilage and the development of food toxins, (2) makes foods less likely to transfer pathogenic microorganisms, (3) generally preserves foods between the time of harvest and consumption, (4) modifies the flavor of the original ingredients and often improves nutritional value [6].

Examples of lactic acid-fermented foods in Asia are given in Table 1. Products in the same category are similar in processing procedures and microorganisms but differ in name and usage per country [1,5].

**Table 1:** Examples of acid-fermented foods in Asia

Product	Country	Major ingredients	Microorganisms	Appearance/ usage
***Rice-wine/beer***				
***Takju***	**Korea**	**rice, wheat**	***Lactic acid bacteria*** ***Saccharomyces cerevisiae***	**Turbid liquid**
***Tapuy***	**Philippine**	**rice, glutinous rice**	***Sacchromyces,*** ***Mucor, Rhizopus, Aspergilus*** ***Leuconostoc*** ***Lb. plantarum***	**Sour, sweet liquid, paste**
***Brem bali***	**Indonesia**	**glutinous rice**	***Mucor indicus, Candida***	**Dark brown liquid sour alcoholic**

***Acid-leavened bread/noodle***				
***Idli***	**India** **Sri Lanka**	**rice** **black gum**	***L. mesenteroides*** ***S. faecalis***	**steamed cake**
***Puto***	**Philippines**	**rice**	***L. mesenteroides*** ***Streptococcus faecalis***	**steamed cake**
***Kichuddok***	**Korea**	**rice**	***yeast***	**steamed cake**
**Mungbean**	**China**	**mungbean**	***L. mesenteroides***	**noodle**
**Starch Noodle**	**Thailand, Korea, Japan**		***Lb. casei,*** ***Lb. cellobiosus*** ***Lb. fermenti***	
***Khanomjeen***	**Thailand**	**rice**	***Lactobacillus sp.*** ***Streptococcus sp.***	**noodle**

***Fermented vegetable***				
***Kimchi***	**Korea**	**Korean cabbage, radish, various vegetables, salt**	***L. mesenteroides*** ***Lb. brevis,*** ***Lb. plantarum***	**salad, side dish**
***Dhamuoi***	**Vietnam**	**cabbage, vegetable**	***L. mesenteroides*** ***Lb. plantarum***	**salad, side dish**
***Dakguadong***	**Thailand**	**mustard leaf**	***Lb. plantarum***	**salad, salt side dish**
***Burong mustala***	**Philippines**	**mustard**	***Lb. brevis*** ***Pediococcus cerevisiae***	**salad, side dish**

***Fermented fish and meat***				
***Sikhae***	**Korea**	**sea water fish** **cooked millet, salt**	***L. mesenteroides*** ***Lb. plantarum***	**side dish**
***Narezushi***	**Japan**	**sea water fish** **cooked millet, salt**	***L. mesenteroides*** ***Lb. plantarum***	**side dish**
***Burong-isda***	**Philippines**	**fresh water fish rice,** **salt**	***Lb. brevis,*** ***Streptococcus sp.***	**side dish**
***Pla-ra***	**Thailand**	**fresh water fish salt,** **roasted rice**	***Pediococcus sp.***	**side dish**
***Balao-balao***	**Philippine**	**shrimp, rice, salt**	***L. mesenteroides*** ***P. cerevisiae***	**condiment**
***Kungchao***	**Thailand**	**shrimp, salt** **sweetened rice**	***P. cerevisiae***	**side dish**
***Nham***	**Thailand**	**pork, galic, salt, rice**	***P. cerevisiae,*** ***Lb. plantarum*** ***Lb. brevis***	**pork meat in banana leaves**
***Nem-chua***	**Vietnam**	**pork, salt, cooked rice**	***Pediococcus sp.*** ***Lactobacillus sp.***	**sausage**

## Discussion

### Rice-wine/beer

Rice-wine is a generic name referring to alcoholic beverages made from cereals, mainly rice, in East-Asia. Traditional alcoholic beverages vary from crystal-clear products to turbid liquid or thick gruels and pastes. Clear products, which are generally called *shaosingjiu* in China, *cheongju* in Korea, and *sake* in Japan, contain around 15% alcohol and are designated as rice-wine, whereas turbid beverages, *takju* (or *maggolli*) in Korea and *tapuy* in the Philippines, contain less than 8% alcohol along with suspended insoluble solids and live yeasts, and are referred to as rice-beer [5].

The process of cereal alcohol fermentation involves a two-step fermentation; solid state fermentation wherein molds grow on raw or cooked cereals, which is called *nuruk*, followed by mashing the *nuruk* with additional cereals to produce alcohol by yeast. The dried and powdered *nuruk* is then mixed with water and stored in a cool place for several days to make the mother brew. During this period, microbial amylases and proteases are produced, which convert the starch present in the cereal raw-materials into sugars. The acid-forming bacteria in *nuruk* then produce organic acids, reducing the pH to below 4.5, which favors the growth of yeast at the later stage of alcohol fermentation. About two to three volumes of cooked grains and water are then added to the mother brew to prepare the first fermentation mash. Upon addition of new cooked grains and water to the mash, the production volume increases while the alcohol concentration and quality of the final product are enhanced. Multiple brews prepared by adding two to nine additions of newly cooked grains to the fermenting mash have been described in the old literatures [7].

**Figure 1:**
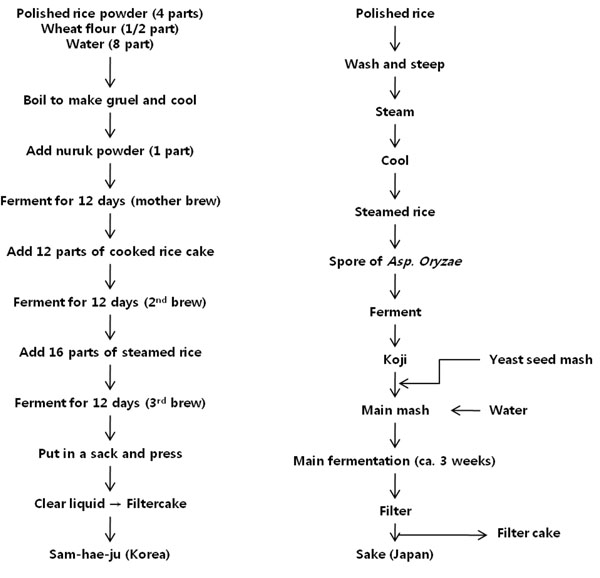
Flow charts for Korean *samhaeju* and Japanese *sake*. Adapted from Lee, 2001[7]

The traditional method of rice-wine brewing was first industrialized by Japanese brewers in the early 20th century, who adopted a pure starter culture, rice *koji*, in combination with manufacturing technology developed in Europe. This production process was later transferred to Korea and China. Industrial production of rice wine involves steaming of polished rice, inoculation of mold, *Aspergillus oryzae* or *Aspergillus kawachii*, and incubation at 25°C-30°C for 2-3 days. The mother brew is made by mixing of the starter culture, *koji*, with yeast seed mash and water, followed by incubation for another 3-4 days at 20°C. The main brew is made by adding ca. 10 volumes of cooked rice and water to the mother brew, followed by fermentation for 2-3 weeks. The fermented mash is then filtered to obtain a clear liquid and aged in a cool place for 1-2 weeks, after which it is filtered again, bottled, and pasteurized [2]. Figure 1 compares the brewing processes of traditional rice-wine (*samhaeju*) and industrial rice-wine (*sake*).

**Figure 2:**
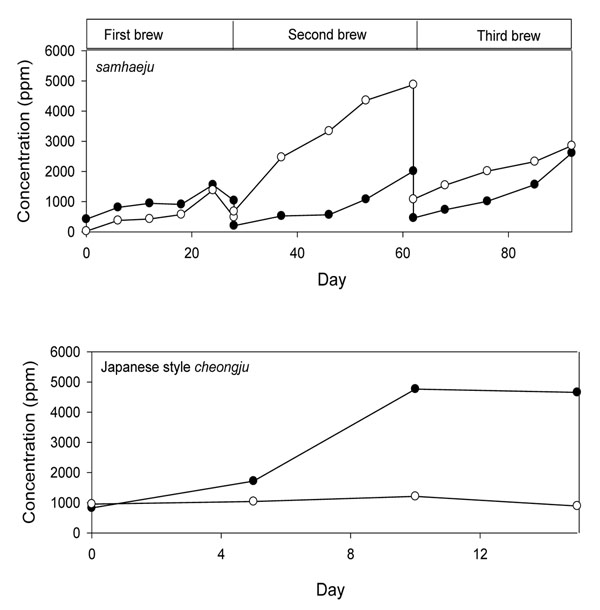
Changes in lactic acid(○) and succinic acid(●) contents during *samhaeju* and c*heongju* brewing. Adapted from Rhee et.al., 2003[2]

Table 2 shows that the changes in microbiota during traditional Korean rice-wine (s*amhaeju*) brewing differ from those of the industrial process (Japanese style *cheongju* or s*ake*), which involves pure culture inoculation in a controlled fermentation process[2]. The contribution of acid-forming bacteria is large in traditional brewing, whereas it is relatively meager in the industrial process. In traditional brewing, lactic acid is the major organic acid produced, whereas industrial rice wine contains mostly succinic acid, which is most likely produced by molds and yeasts (Figure 2)[2]. Moreover, traditional rice wine contains significantly higher concentrations of ethylacetate (75 ppm) and lower concentrations of n-propanol (70 ppm), isobutanol (125 ppm), and isoamylalcohol (210 ppm) relative to industrial products [2]. The differences in microbiota and concentrations of corresponding flavor compounds result in differential sensory qualities, wherein traditional rice-wine has a deep and bounty-like flavor and industrial ones are characterized by a more simple and light flavor.

**Table 2:** Changes in concentrations of microorganisms during *samhaeju* and *cheongju* brewing.

Microorganism (CFU/mL)		***samhaeju***			Japanese style *cheongju*	
	
	Batch	1^st^ brew	2^nd^ brew	3^rd^ brew	Mother brew	Main brew
**Mold**	**A**	**4.2 x 10^5^**	**< 10^2^**	**ND**	**ND**	**ND**

	**B**	**9.8 x 10^4^**	**< 10^2^**	**ND**	**ND**	**ND**
	
	**C**	**3.9 x 10^5^**	**< 10^2^**	**ND**	**ND**	**ND**

**Acid forming bacteria**	**A**	**3.1 x 10^5^**	**8.9 x 10^7^**	**8.6 x 10^4^**	**ND**	**< 10^2^**
	
	**B**	**7.5 x 10^6^**	**3.3 x 10^6^**	**6.3 x 10^4^**	**ND**	**< 10^2^**
	
	**C**	**3.3 x 10^5^**	**8.5 x 10^7^**	**7.5 x 10^4^**	**ND**	**< 10^2^**

**Yeast**	**A**	**< 10^2^**	**1.8 x 10^6^**	**1.8 x 10^6^**	**3.2 x10^7^**	**2 x 10^6^**
	
	**B**	**< 10^2^**	**2.4 x 10^6^**	**1.6 x 10^6^**	**1.9 x 10^7^**	**2.5 x 10^7^**
	
	**C**	**< 10^2^**	**4.6 x 10^6^**	**1.4 x 10^6^**	**2.6 x10^6^**	**3.1 x 10^6^**

Adapted from Rhee et.al., 2003[2]

### Acid-fermented bread and noodle

Lactic acid fermentation of bread dough improves the keeping quality and flavor of the baked products. It also enhances the palatability of bread made from low grade flours and under-utilized cereals. Acid-fermented breads and pancakes are an important staple food for people in Africa and some parts of Europe and Asia [8].

**Figure 3:**
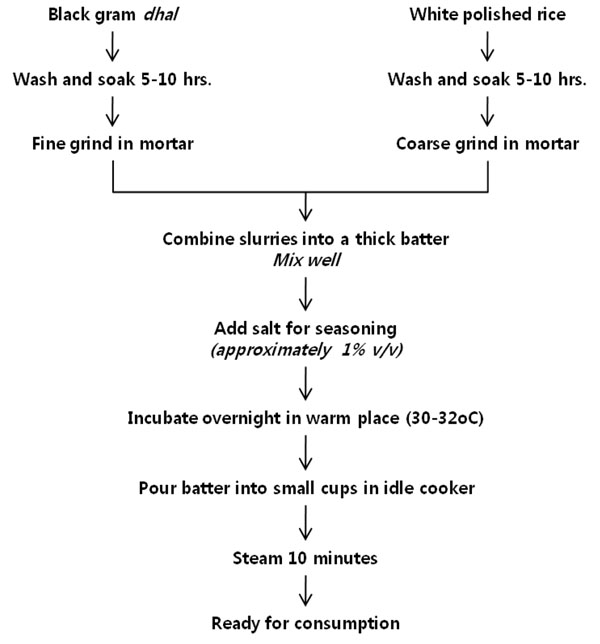
Flow chart for *idli* production. Adapted from Steinkraus, 1983[6]

Large quantities of acid-leavened bread and pancakes are consumed daily in India, Sri-Lanka, Pakistan, Nepal, Sikkim, Tibet, and neighboring countries. *Idli*, *dosa*, and *dhokla* are produced primarily in south India and Sri Lanka. *Idli* is a small, white, acid-leavened and steamed cake prepared by bacterial fermentation of a thick batter made from carefully washed and coarsely ground rice as well as dehulled and finely ground black gram *dhal.Dosa* batter is very similar to *idli* batter, except that the rice and black gram are both finely ground. Following fermentation, *dosa* is quickly fried as a thin, fairly crisp pancake and eaten directly. *Dhokla* is similar to *idli* except that dehulled Bengal gram *dhal* is used in place of black gram *dhal*. The fermented batter is poured into a greased pie tin and steamed in the open rather than in a covered *idli* steamer. Figure 3 shows the flow chart of *idli* production [6].

*L. mesenteroides* and *Streptococcus faecalis* are developed concomitantly with soaking and then continue to multiply following grinding. Both genera eventually reach higher than 1x10^9^ cells/gram of the finished batter [9]. *L. mesenteroides* is considered to be essential for leavening of the batter, and it is also considered to be responsible for acid production in *idli*, *dosa*, and related products, together with *S. faecalis*.

These organisms appear to be present in the raw-material ingredients, and therefore it is generally not required to add them as inoculum. Aerobic contaminants that are usually present in the raw-materials are eliminated partly by careful washing of the ingredients and partly by the acidic conditions generated by the fermentation. However, Batra and Millner [10] isolated *Torulopsis candida* and *Trichosporon pulluans* from *idli* batter and prepared authentic *idli* only by the joint action of both yeasts in the mixture. Both *T. pullulans* and *T. candida* provide characteristic acidity, whereas *T. candida* also produces gas during fermentation.

**Figure 4:**
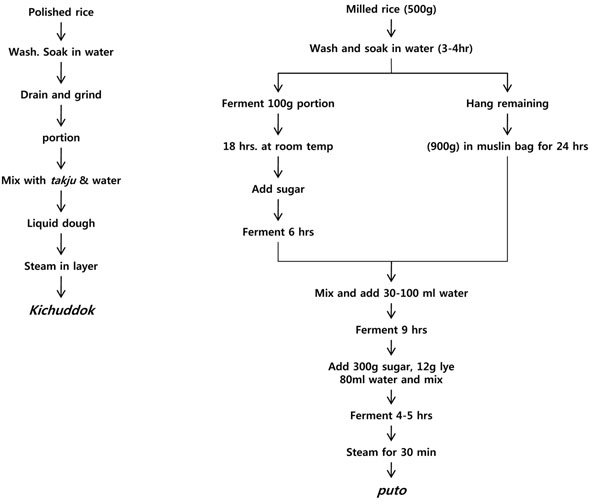
The processing procedures of *kichuddok* and *puto*. Adapted from Lee, 2001[7]

Leavened bread-type foods are not traditional staples in East Asia, although yeast-fermented breads are commonly used today. Chinese people have traditionally used steamed bread, *mantou*, which is prepared by steaming yeast-leavened wheat dough, often filled with sweets, meats, and vegetables. Other types of breads are prepared primarily by acid fermentation of rice flour dough, including Korean *kichuddok* and Philippine *puto*. These products are leavened, steamed rice cakes that are similar to Indian *idli*, except that they do not contain any legumes. *Puto* is special in the sense that it is prepared from year-old rice and its batter is neutralized at the mid-point of fermentation. Figure 4 shows the processing procedures of *kichuddok* and *puto*[*7*].

*Kichuddok* is prepared at the household level and consumed less frequently at special occasions in Korea, whereas *puto* is normally consumed as a breakfast and snack in the Philippines. *Puto* is a common food for the lower-income group, but special types with added cheese, eggs, etc., are consumed as delicacies by the higher-income group. In a number of Philippine towns, preparation of puto is an important cottage industry [7].

Thai rice-noodle, *khanom-jeen*, is also made from acid-fermented rice [11]. Soaked rice is drained and fermented for at least 3 days before grinding, and *Lactobacillus* species and *Streptococcus* species are involved in the acid fermentation. Acid-fermented porridges, such as *ogi* and *uji* in African countries, are not common in the Asia-Pacific region.

Most Asian countries produce mungbean starch, and mungbean starch noodles are dietary staples of the Chinese. The process for manufacturing mungbean starch involves acidic bacterial fermentation, in which the mungbeans are hydrated by soaking in water inoculated with 12-hr steep water from a previous fermentation to ensure acidification. The principal microorganisms found in the steep water are *L. mensenteroides*, *Lactobacillus casei*, *Lactobacillus cellobiosus*, and *Lactobacillusfermentum*. Lactic acid fermentation, which reduces the pH from 6.0 to about 4.0 protects the starch granules from spoilage and putrefaction that would otherwise occur in non-inoculated ground bean slurries [7].

### Acid-fermented fish and meat

**Figure 5:**
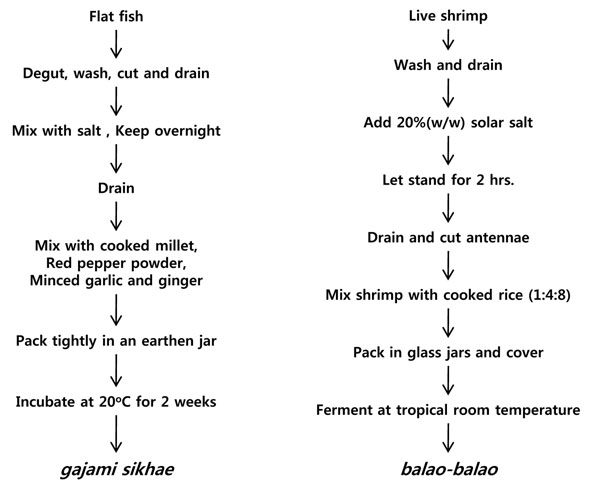
The processing procedure of Korean *sikhae* and Philippine *balao-balao*. Adapted from Lee, 2001[7]

The storage life of perishable fish and meats can been extended by acid-fermentation with added carbohydrates and salts. Both freshwater and seawater fish are preserved by this method. Rice, millet, flour, and even syrup or sugar are all used as carbohydrate sources. Millet is used as the main carbohydrate source in Northeastern countries, whereas in Southeastern countries, rice is commonly used as a carbohydrate source. The organic acids produced from the added carbohydrates in combination with salt control the extent of acid fermentation and keep the quality of the product [12]. Figure 5 illustrates the processing procedure of Korean *sikhae* and Philippine *balao-balao*[7].

**Figure 6:**
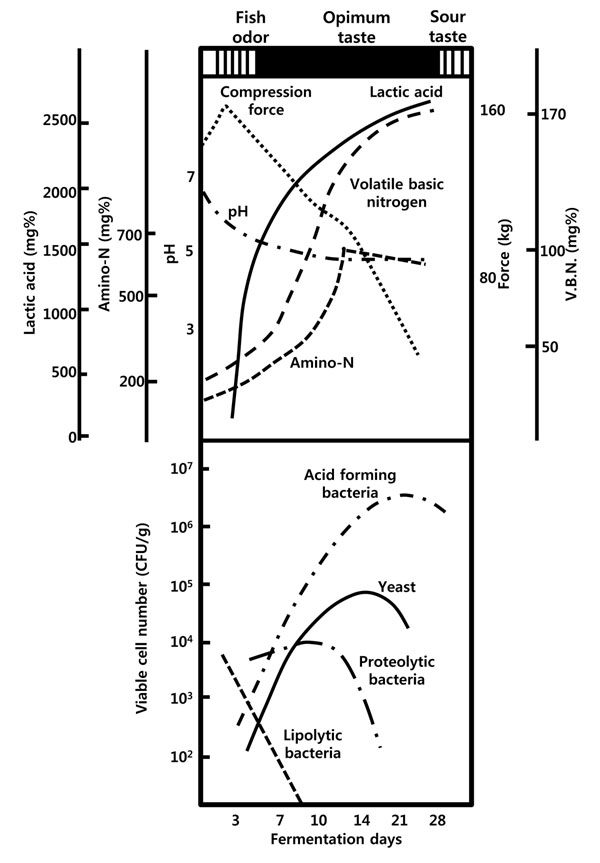
The microbial and biochemical changes during *sikhae* fermentation . Adapted from Lee, 1994[1]

Figure 6 shows the microbial and biochemical changes of a typical lactic acid-fermented fish product, *sikhae*, incubated at 25°C[1]. The pH decreases rapidly during the first 3-5 days from 6.5 to below 5.0, whereas the texture softens within 3-4 days. The amino-N concentration increases steadily for 14 days, concomitant with the generation of optimum flavor. The number of lipolytic bacteria decreases rapidly during the initial stage of fermentation, whereas the number of proteolytic bacteria increases until 12 days of fermentation, followed by a rapid decrease thereafter. The typical acid-forming bacteria rapidly increase in number, becoming the predominant microbes within 1 week of fermentation and reaching their maximum density after 16 days. Typically, flavor deterioration in these products is associated with rapid growth of yeast [13]. Important bacteria for the lactic acid fermentation of *sikhae* have been identified as *L.mesenteroides* and *Lactobacillus plantarum*[*14*]. The role of these acid-forming bacteria in the preservation of fish has been established, but an even more important contribution is their ability to produce acceptable flavor during fermentation of the product.

### Acid-fermented vegetables

Acid-fermented vegetables are important sources of vitamins and minerals. *L. mesenteroides* has been found to be important in the initiation of fermentation of many vegetables, i.e. cabbages, beets, turnips, cauliflower, green beens, sliced green tomatoes, cucumber, olives, and sugar beet silages[1]. In vegetables, *L. mesenteroides* grows rapidly and produces carbon dioxide and acids that quickly lower the pH, thereby inhibiting the development of undesirable microorganisms and the activity of their enzymes as well as preventing unfavorable softening of the vegetables. The carbon dioxide produced replaces air and provides anaerobic conditions that favor stabilization of ascorbic acid and the natural colors of the vegetables. *L. mesenteroides* converts glucose to approximately 45% levorotatory D-lactic acid, 25% carbon dioxide, and 25% acetic acid and ethyl alcohol. Moreover, fructose is partially reduced to mannitol, which subsequently undergoes secondary fermentation (see below) to yield equimolar quantities of lactic acid and acetic acid. The combination of acids and alcohol are conducive to the formation of esters, which impart desirable flavors [1]. Overall, the initial growth of *L. mesenteroides* leads to modification of the environment that favors the growth of other lactic acid bacteria. Secondary fermentation in these processes, especially by homofermentative *Lactobacillus* species, leads to further reduction of the pH and ultimately growth of *L. mesenteroides.*

*Kimchi* fermentation is the Korean method of preserving the fresh and crispy texture of vegetables during the winter when fresh vegetables are not available. Almost all kinds of vegetables can be made into *kimchi*; cabbages, radish, cucumber, Welsh onion leaves, and mustard leaves are the popular main ingredients. The name of each particular *kimchi* is based on the main ingredients: cabbage *kimchi* (*baechukimchi*), radish *kimchi*, cucumber *kimchi*, etc. Minor ingredients such as garlic, red pepper, green onion, ginger, and salt are also added. Fermented fish products and other seasoning agents are optional. *kimchi* has a sour, sweet, and carbonated taste and is usually served cold [7]. It is a side dish that is commonly served with cooked rice and soup.

**Figure 7:**
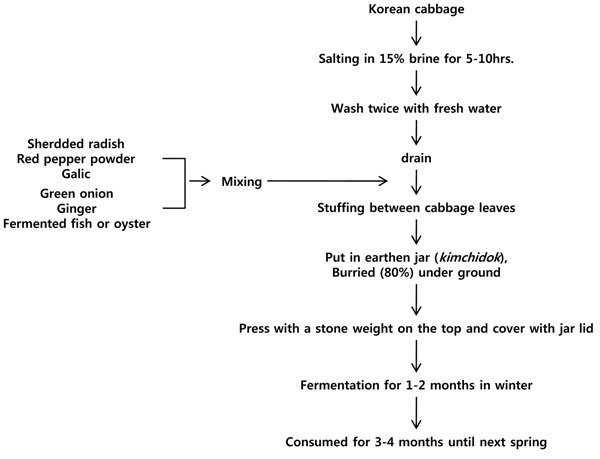
Flow chart of *kimchi* (winter *baechukimchi*) making process. Adapted from Lee, 2001[7]

**Figure 8:**
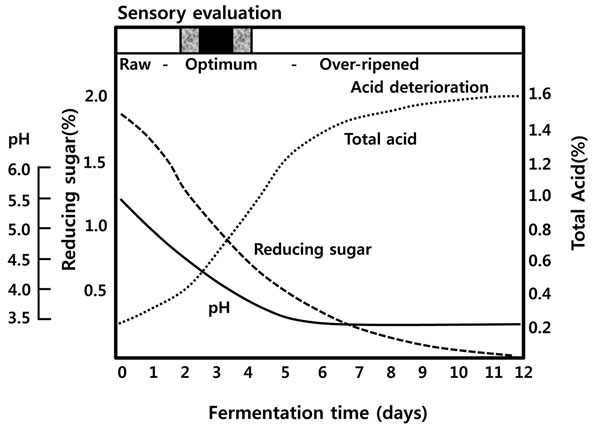
Changes in pH, acidity and reducing sugar content during *kimchi* fermentation. Adapted from Lee, 2001[7]

**Figure 9:**
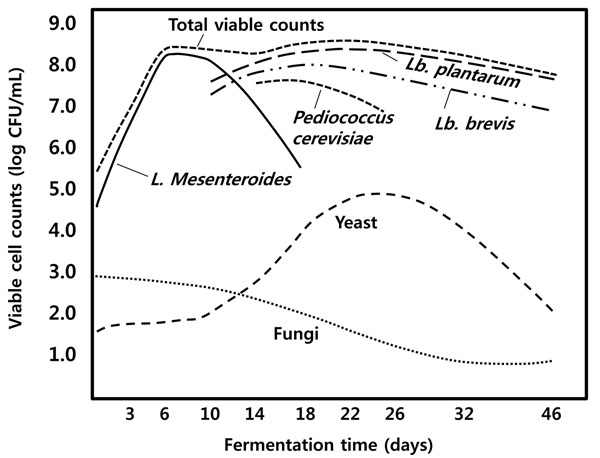
Cahnges in microflora during *kimchi* fermemtation at 14°C (3.5% NaCl). Adapted from Lee, 1994[1]

One recipe for cabbage *kimchi* may include 100 g of Korean cabbage, 2 g of garlic, 2 g of green onion, 2 g of red pepper powder, and 0.5 g of ginger, and the optimum salt content of the product is 3% (Figure 7)[7]. For preparation, fresh cabbage is cut in half or shredded, soaked in brine of approximately 10% salt concentration overnight (or 15% salt brine for 5-10 hours), and then washed and drained. The minor ingredients are chopped and mixed, with shredded radish stuffed between the salted cabbage leaves. The *kimchi* is then packed in an earthen jar, *onggi* or *dok*, buried in the ground, and pressed with a stone in order to submerge them in the juice. Winter *kimchi* is fermented for 1-2 months and consumed for 3-4 months until the end of the spring season. Figure 8 shows the biochemical changes in *kimchi* during fermentation [7]. Optimum taste is attained when the pH and acidity reach approximately 4.0-4.5 and 0.5-0.6 (lactic acid equivalent %), respectively. Vitamin C content is maximal at this point [15]. At a higher fermentation temperature, the ripening time is decreased; *kimchi* ripens in 1 week at 15 and in 3 days at 25 [7]. Before-ripening, *L. mesenteroides* is the dominant microorganism, whereas *Lactobacillus* species are the major organisms in over-ripened *kimchi* (Figure 9) [1].

The dominant species of *Lactobacillus* in the later stages of *kimchi* fermentation vary according to the fermentation temperature; *Lb. plantarum* and *Lactobacillus brevis* dominate fermentations carried out at 20-30 while *Lactobacillus maltaromicus* and *Lactobacillus bavaricus* dominate at 5-7. *Lb. plantarum* is homofermentative and is the highest acid-producing species of this group, yielding three or four times higher DL-lactic acid content than *Leuconostoc* species [*1*]. Low temperature is preferred for *kimchi* fermentation to prevent the production of high amounts of lactic acid and over-ripening as well as to extend the period of optimum taste.

Recently, the genome probing DNA chip (GPM) method was applied to identify and monitor microbial behavior during fermentation. Over 100 species of microorganisms were identified in *kimchi* fermentation [16]. Among these, *Weisella confuse*, *Leuconostoc citreum*, *Lactobacillus curvatus*, *Lactobacillus sakai*, *and Lb. fermentum* were identified as the important microorganisms.

In an inoculation test, the strong anti-pathogenic activity of *kimchi* was demonstrated (Table 3)[7]. *Clostridium perfringens* disappeared after 2 days of *kimchi* fermentation, *Staphylococcus aureus* and *Salmonella typhymurium* after 4 days, and *Listeria monocytogenes*, *Vibrio parahaemolyticus*, and *Escherichia coli* after 5 days; however, the number of lactic acid bacteria increased from 10^5^ to 10^8^[17,7]. The inhibitory effects of *kimchi* ingredients, for example garlic, and the fermentation metabolites (organic acids) are well known. Garlic has anti-microbial activity specific to some pathogens while simultaneously having no effect on lactic acid bacteria [14].

**Table 3:** Changes in concentrations of intestinal pathogens in *kimchi* during fermentation at 20°C ((CFU/mL)).

Fermentation(days)	0	1	2	3	4	5	6	7
**pH**	5.44	5.12	4.11	3.86	3.76	3.70	3.66	3.63
***Cl. perfringens***	4.3×10^4^	2.7×10^2^	-	-	-	-	-	-
***Staph. aureus***	2.9×10^4^	4.5×10^4^	2.8×10^3^	5.0×10	-	-	-	-
***S. typhimurium***	3.6×10^4^	2.2×10^4^	5.8×10^3^	1.1×10^2^	-	-	-	-
***L. monocytogenes***	6.3×10^4^	3.7×10^4^	4.5×10^3^	2.6×10^2^	4.0×10	-	-	-
***V. parahaemolyticus***	2.3×10^4^	2.1×10^4^	7.3×10^3^	5.5×10^2^	9.0×10	-	-	-
***E. coli***	5.2×10^4^	3.3×10^4^	2.9×10^3^	3.3×10^2^	3.0×10	-	-	-
**Lactic acid bacteria**	2.0×10^5^	7.3×10^6^	2.8×10^8^	5.7×10^8^	6.1×10^8^	5.6×10^8^	5.8×10^8^	6.0×10^8^
**Total bacteria**	4.4×10^4^	3.6×10^7^	9.3×10^8^	1.2×10^9^	1.5×10^9^	1.4×10^9^	1.6×10^9^	1.4×10^9^

Adapted from Lee, 2001[7]

Several strains of microorganisms that produce bacteriocin have been isolated from *kimchi*. *Enterococcus faecium* in *kimchi* has a broad spectrum of bacteriocin activities, and several *Lactobacillus* species have been shown to produce anti-microbial compounds [17,7]. As an example, a heat- and pH-stable bacteriocin, kimchicin GJ7, produced by *L. citreum* GJ7 was isolated. Notably, the presence of a bacteriocin-sensitive strain, *Lb. plantarum*, was shown to act as an environmental stimulus to activate the production of kimchicin GJ7 by *L. citreum*[18]. Furthermore, improved quality and shelf-life of *kimchi* by fermentation using an induced bacteriocin-producing strain as a starter were previously observed [18]. Recently, a new antifungal compound, 3,6-bis(2-methylpropyl)-2,5-piperazinedion (molecular mass of 226 kDa), was identified as being produced by a *Lb. plantarum* strain obtained from *kimchi*[*19*], illustrating that the anti-microbial pallet produced during *kimchi* fermentation may exceed the anti-bacterial activities. Overall, the combination of organic acids and anti-microbial compounds produced during fermentation and the anti-microbial activity of the ingredients regulate the microbiota found in *kimchi*, and it controls the growth of pathogenic microorganisms without costly treatments and packaging.

The physiological effects of *kimchi* ingredients and their metabolites have been studied extensively [20,7]. The anti-tumor activities of cabbage and garlic have been reported by many investigators [21], whereas extracts of red pepper powder have been shown to exert inhibitory effects against aflatoxin B_1_-mediated mutagenesis. Additionally, *kimchi* contains sufficient concentrations of fiber to prevent constipation and colon cancer, as well as exerting prebiotic effects. Finally, the probiotic effect of lactic acid bacteria in *kimchi* (grown to 10^8^/mL) may assist in digestive and intestinal functions [22] (Table 4)[7].

Thus, *kimchi* is a synbiotic food consumed widely in Korea [23]. In addition to these physiological effects, the salty taste, fresh carbonated sensation, and crispy texture of *kimchi* has made it the most favored and indispensable food for Koreans. According to a recent national food consumption survey, a single male Korean adult consumes 50-100 g/day of *kimchi* in the summer and 100-200 g/day in winter.

**Table 4:** Biologically active compounds in *kimchi*.

Chemical compounds	Occurrence	Possible effect
**Benzylisothiocyanate**	**Chinese cabbage**	**antibiotic**
**Indol compound**	**allium vegetable**	**anticarcinogenic**
**Thiocyanate, Flavonoid**	**Red pepper**	**immune stimulant**
**sistosterol**	**Chinese cabbage**	**reducing the cholesterol level**
**Diallysulfide**	**allium vegetable**	**anticarcinogenic**
**Diallytrisulfide**		**antioxidant**
**Diallymethylsulfide**		**fibrinolytic**
**Gingerrol**	**ginger**	**antibiotic**
**Gingerin**		**fibrinolytic**
**Capsaicin**	**red pepper**	**laxative**
		**secretion of neuropeptides**
**Lactic acid bacteria**	***Kimchi***	**antagonistic**
**Bacteriocine**	***Kimchi***	**antibiotic**
**L-(+) lactic acid**	***Kimchi***	**modulation of T-cell function**
**Acethylcholine**	***Kimchi***	**laxation**
**Dextran**	***Kimchi***	**laxation**
**γ-aminobutyric acid**	***Kimchi***	**laxation**
**Acetate**	***Kimchi***	**antibiotic**

Adapted from Lee, 2001[7]

### Adaptation of lactic acid bacteria in human food cycle

The bacteria isolated from *kimchi* are identified in Bergey's Manual, but their physiological characteristics seldom match exactly with those characterized in the Manual. *L. mesenteroides* and *Lb. barvaricus* isolated from *kimchi* show many discrepancies in sugar fermentation and vitamin requirements. All *Leuconostoc* species isolated from *kimchi* can grow at pH levels below 4.8 as well as in media containing 7% ethanol or 6.5% NaCl [7]. An interesting observation is that *L. mesenteroides* subspecies show tolerance in artificial digestive fluid at pH 3.0 and also grow in media containing 10% or 40% bile [24]. These properties are similar to those of intestinal microorganisms, such as *Lactobacillus acidophilus* and *Lb. casei*, as well as faecal microbial strains. These observations suggest that the major microorganisms in *kimchi* have adapted to the special environment of Korea as a part of the food cycle from the soil to vegetables, to *kimchi*, and then to the human intestine, faeces, and the soil again. Adaptations of microorganisms to special environmental conditions have been reported in other fermented foods, i.e. *L. mesenteroides* in cane juice, *Leuconostoc. oenos* in grape juice, *Pediococcus. halophilus* in soy sauce, and the above-mentioned *L. mesenteroides* in *sikhae*[7].

## Conclusions

Lactic acid bacteria play important roles in many Asian fermented foods, especially in non-dairy fermented vegetable products. The probiotic functions of lactic acid bacteria in non-dairy fermented foods in Asia have not been fully investigated. *L. mesenteroides* present in *kimchi*, for example, probably have probiotic effects. In fact, Koreans who travel overseas for several days without *kimchi* often experience uncomfortable stomach symptoms and poor digestion. More research is needed to identify the lactic acid bacteria in Asian fermented foods and their physiological functions in the human diet.

## Competing interests

The authors declare that they have no competing interests.

## Authors' contributions

Sook Jong Rhee – assisted in collection of data and editing of the review paper.

Jang-Eun Lee – assisted in collection of data and editing of the paper.

Cherl-Ho Lee – principal researcher and corresponding author.
